# Claudin-3 is expressed at the *macula densa* and is associated with altered glomerular filtration in claudin-3 knockout mice

**DOI:** 10.3389/fphys.2026.1869073

**Published:** 2026-07-24

**Authors:** Alexandra Chassot, Eric Feraille, Ali Sassi

**Affiliations:** Department of Cellular Physiology and Metabolism, Faculty of Medicine, University of Geneva, Geneva, Switzerland

**Keywords:** claudin-3, glomerular filtration rate, *macula densa*, NKCC2, nNOS, sodium transport, tight junction

## Abstract

Claudin-3 is a tight junction protein expressed in the distal nephron, notably in the thick ascending limb of Henle’s loop and the distal convoluted tubule, where it acts as an aldosterone-regulated paracellular barrier to sodium and chloride transport. Here, we demonstrate that claudin-3 knockout (KO) mice exhibit an unexpected diet-dependent alteration in glomerular filtration rate (GFR): under a normal sodium (NS) diet, GFR decreases significantly, whereas under a low sodium (LS) diet, GFR increases paradoxically compared with wild-type controls. To identify the underlying mechanism, we examined claudin-3 expression in the *macula densa*, the specialized epithelial structure located at the junction between the thick ascending limb and the distal convoluted tubule that governs tubuloglomerular feedback (TGF). Immunofluorescence analysis revealed that claudin-3 colocalizes with neuronal nitric oxide synthase (nNOS) and the Na^+^-K^+^-2Cl^-^ cotransporter (NKCC2) in a distinct cluster of cells characterized by densely packed nuclei, positioned in direct contact with the glomerulus and immediately adjacent to an NCC-positive distal convoluted tubule, while remaining itself NCC-negative. Notably, within the tubule containing the *macula densa*, nNOS expression was strictly confined to *macula densa* cells, clearly distinguishing them from the surrounding tubular epithelium. These results identify claudin-3 as a previously unrecognized component of *macula densa* biology and suggest a role for claudin-3 in the control of GFR possibly *via* the tubuloglomerular feedback.

## Introduction

Renal sodium transport regulation is essential for maintaining electrolyte balance, extracellular fluid homeostasis, and blood pressure. GFR determines the sodium load delivered to the kidney tubule and is tightly controlled by TGF, a mechanism through which the *macula densa* senses luminal NaCl concentration and adjusts afferent arteriolar tone accordingly (Schnermann and Levine, 2003; Castrop et al., 2010; Peti-Peterdi and Harris, 2010).

The *macula densa* is a specialized plaque of epithelial cells located at the transition between the thick ascending limb of Henle’s loop (TAL) and the distal convoluted tubule (DCT), often considered as a specialized segment of the early DCT epithelium in direct proximity to the glomerulus. It is characterized by densely packed nuclei, the expression of neuronal nitric oxide synthase (nNOS) and the Na^+^-K^+^-2Cl^-^ cotransporter (NKCC2), and the absence of Na^+^-Cl^-^ cotransporter (NCC) expression within the tubule containing the *macula densa*, which clearly distinguishes *macula densa* cells from the surrounding tubular epithelium (Bachmann et al., 1995; Obermüller et al., 1996; Bell et al., 2003).

Claudins are tight junction proteins that form the structural and functional backbone of epithelial tight junctions and critically determine paracellular ion selectivity (Tsukita et al., 2001; Günzel and Yu, 2013). We recently demonstrated that claudin-3 is expressed along the distal nephron, including the TAL, DCT, connecting tubule, and collecting duct, where it functions as an aldosterone-regulated paracellular barrier to sodium and chloride transport (Sassi et al., 2026a). In the collecting duct, claudin-3 deficiency induces compensatory upregulation of ENaC subunits as well as claudins-4, -8, and -10 under low sodium diet, maintaining normal plasma electrolyte levels (Sassi et al., 2026a). Here, we demonstrate that under normal sodium diet, claudin−3-deficient mice display a reduction in GFR, which represents the only renal phenotype observed under basal condition in these mice. This is observed in the absence of detectable alteration in other major renal functional parameters. Under low sodium diet, these mice exhibit a paradoxical increase in GFR, revealing for the first time a striking diet−dependent alteration of GFR in claudin−3 KO mice. This newly identified bidirectional phenotype provides a plausible explanation for the enhanced adaptive responses previously described in claudin-3–deficient mice under low sodium diet, including ENaC and claudin species upregulation (Sassi et al., 2026a). These findings are consistent with an alteration of TGF, leading us to hypothesize that claudin−3 is expressed in the *macula densa*, where it may contribute to the regulation of TGF signaling and GFR.

## Materials and methods

### Animals and experimental diets

The generation of claudin-3 knockout (KO) mice, dietary protocols, and ethical approvals have been described in detail previously (Sassi et al., 2026a). Briefly, homozygous claudin-3 KO mice and wild-type littermates on a C57BL/6 background were maintained on either a normal sodium, normal potassium (NSNK; 0.18% Na^+^) or a low sodium, normal potassium diet (LSNK; 0.01% Na^+^) for 7 days. All experiments were performed in male mice aged 8–12 weeks during the light phase.

For urine collection, mice were acclimated to Tecniplast metabolic cages for 24 h. Following the acclimation period, urine was collected over the subsequent 24 h period. Urinary electrolyte measurements were performed at the Renal Function Investigation Core Facility, Cordeliers Research Center (Paris, France). Sodium concentration was determined using a flame photometer (M420), while chloride concentration and urine osmolality were measured at the same facility according to standard laboratory procedures.

Blood samples were collected from anesthetized mice *via* the venous sinus using heparinized capillary tubes to prevent coagulation. Samples were immediately analyzed using the Epoc Blood Analysis System (Siemens Healthineers), according to the manufacturer’s instructions, allowing rapid and reliable quantification of plasma electrolytes and related blood parameters.

### Transcutaneous GFR measurement

GFR was measured by transcutaneous FITC-sinistrin clearance, as previously described (Schreiber et al., 2012; Scarfe et al., 2018; Dizin et al., 2020; Arnoux et al., 2025). Briefly, mice were anesthetized with isoflurane, and a miniaturized fluorescence detector (Mannheim Pharma and Diagnostics, Mannheim, Germany) was mounted on the shaved dorsal skin. Background fluorescence was recorded for 5 minutes prior retro-orbital injection of FITC-sinistrin (5mg/100g body weight; Mannheim Pharma and Diagnostics, Germany).

Following tracer injection, transcutaneous fluorescence was continuously recorded for 1.5 h in conscious freely moving mice. The elimination kinetics of FITC-sinistrin were analyzed using MPD Lab software (Mannheim Pharma and Diagnostics, Germany). GFR was calculated from the plasma elimination half-life (t_1/2_) of FITC-sinistrin using the validated conversion factor provided by the manufacturer, according to the equation: GFR (µL/min) = 14616.8/t_1/2_ (min). For normalization to body weight, GFR values were additionally expressed per 100 g body weight using the following calculation: normalized GFR (µL/min/100 g body weight) = absolute GFR (µL/min) × 100/body weight (g).

### Immunofluorescence

Immunofluorescence staining was performed as previously described (Sassi et al., 2026a). Briefly, kidneys were fixed, dehydrated, and paraffin-embedded before sectioning at 5 µm thickness. Antigen retrieval was performed using Tris-EDTA buffer (10 mM Tris base, 1 mM EDTA solution, 0.05% Tween 20, pH 9.0). Sections were permeabilized with 0.2% Triton X-100 in PBS and blocked for 1 hour with 10% normal goat serum in TBST.

Primary antibodies were applied overnight at 4 °C, followed by incubation with the appropriate fluorescent secondary antibodies. Nuclear staining was performed using DAPI where indicated. Fluorescence images were acquired using a Zeiss Axio Imager M2 microscope.

Because several primary antibodies used to identify *macula densa* markers were raised in the same host species, and to avoid potential antibody competition or cross-reactivity that could interfere with signal interpretation, serial paraffin sections were used to analyze multiple markers in adjacent sections of the same anatomical region. This approach enabled reliable assessment of claudin-3, neuronal nitric oxide synthase (nNOS), the Na^+^-K^+^-2Cl^-^ cotransporter (NKCC2), and the Na^+^-Cl^-^ cotransporter (NCC) expression patterns within the *macula densa* region.

The specificity of the claudin-3 antibody was previously validated by both Western blot and immunofluorescence using claudin-3 KO kidney tissue (Sassi et al., 2026a). All antibodies used in this study are listed in **Table 1**.

**Table 1:** Antibodies used for immunofluorescence.

Name	Species	Dilution for IF	Supplier	Cat. number
Claudin-3	Rabbit	1/50	Abcam	ab15102
nNOS	Mouse	1/50	BD Biosciences.	N31020
NKCC2	Rabbit	1/800	Prof. J. Loffing	(Moser et al., 2021)
NCC	Rabbit	1/800	Prof. J. Loffing	(Sorensen et al., 2013)

### Statistical analysis

Statistical analyses were performed using Prism version 10.6.1 (GraphPad Software, San Diego, CA, USA). Comparisons among multiple groups were conducted using two-way ANOVA (genotype x diet), followed by Tukey’s *post hoc* test. Data are presented as mean ± SD. A p-value < 0.05 was considered statistically significant.

## Results

### Claudin-3 deficiency alters GFR in a dietary sodium-dependent manner

**Figure 1:**
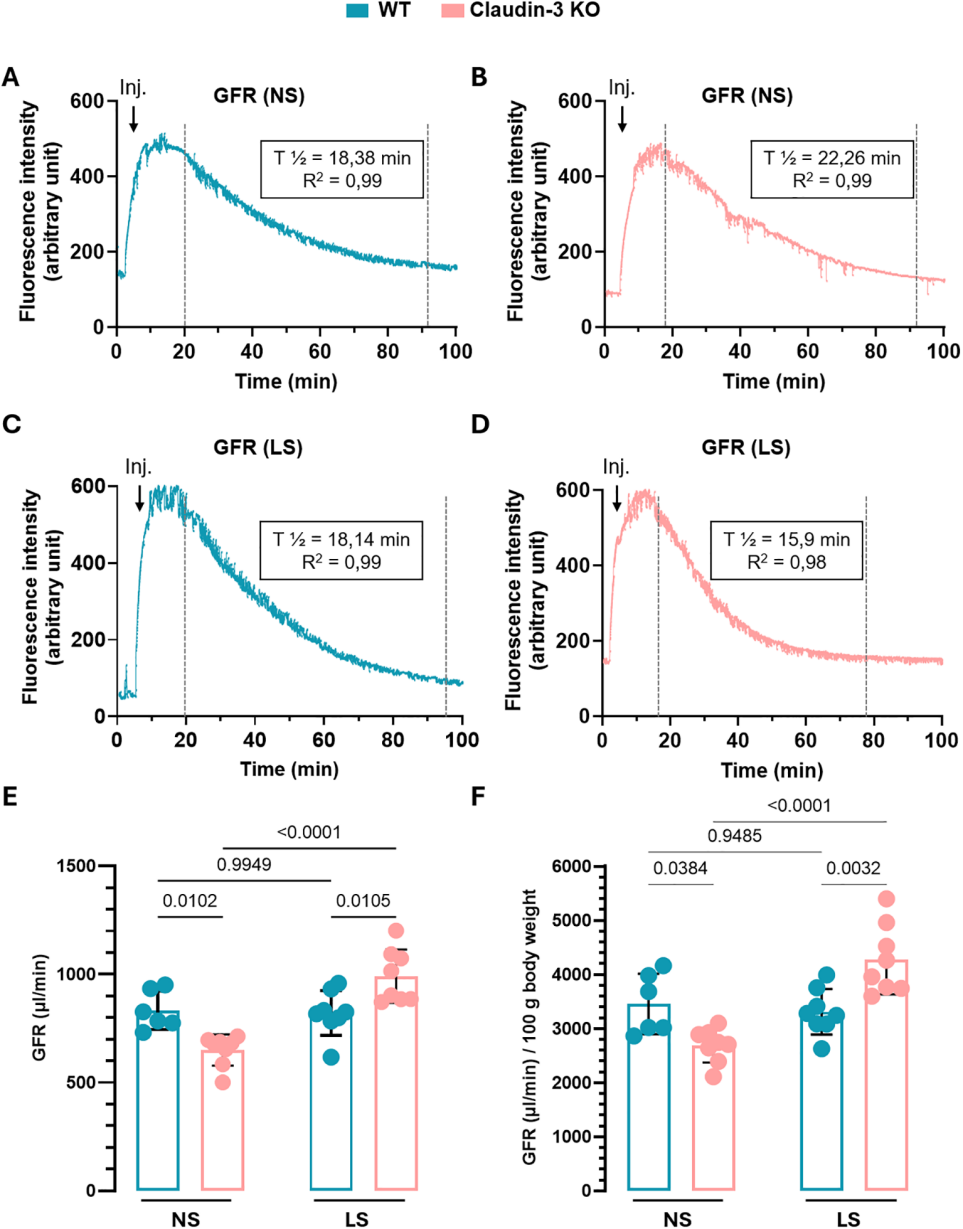
Diet-dependent alteration of GFR in claudin-3 knockout mice. GFR was measured by transcutaneous FITC-sinistrin clearance in wild-type (WT) and claudin-3 knockout (KO) mice maintained on a normal-sodium (NS) or low-sodium (LS) diet for 7 days. Representative GFR tracings are shown for WT and claudin-3 KO mice under NS conditions **(A, B)** and LS conditions **(C, D)**. Tracings include the baseline period, FITC-sinistrin administration, and the elimination phase used for GFR calculation. Arrows labeled “Inj” indicate the time of FITC-sinistrin injection. Vertical dashed lines indicate the beginning and end of the elimination phase used to determine FITC-sinistrin half-life. The elimination half-life (t_1/2_) and goodness-of-fit (R^2^) are indicated for each representative tracing. **(E)** Absolute GFR and **(F)** GFR normalized to 100 g body weight in WT and KO mice under NS and LS conditions. Statistical analysis was performed using two-way ANOVA (genotype x diet), followed by Tukey’s multiple comparisons test. Significant effects were observed for diet [**(E)**, p = 0.0001; **(F)**, p = 0.0005] and genotype x diet interaction [**(E)**, p < 0.0001; **(F)**, p < 0.0001], whereas the main effect of genotype was not significant [**(E)**, p = 0.8469; **(F)**, p = 0.5874]. *Post-hoc* comparison p-values are indicated in the image. Data are mean ± SD. Sample sizes: except for WT-NS (n = 6), all groups had n = 8.

**Figure 2:**
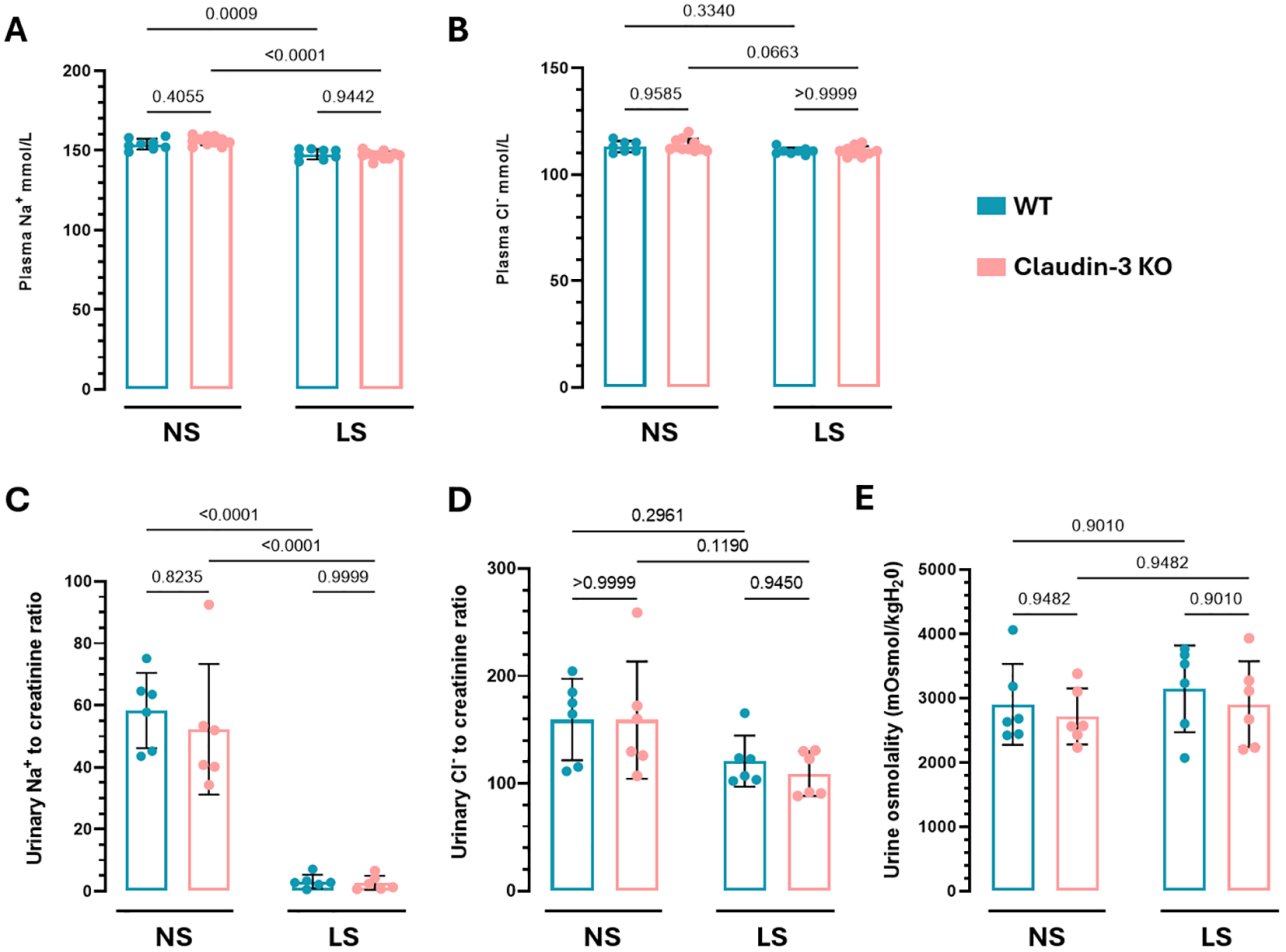
Plasma and urinary ion concentrations and urine osmolality in WT and claudin-3 KO mice under normal-salt (NS) and low-salt (LS) diet for 7 days. Plasma analysis from venous blood showing sodium (Na^+^) **(A)** and chloride (Cl^-^) **(B)** concentrations in WT and claudin-3 KO mice under NS and LS conditions. Urine was collected over a 24-h period, and urinary sodium (Na^+^) **(C)**, chloride (Cl^-^) **(D)**, and urine osmolality **(E)** were measured. Data were analyzed using two-way ANOVA (genotype x diet) followed by Tukey’s multiple comparisons test. A significant effect of diet was observed for plasma Na^+^ [**(A)**, p < 0.0001], plasma Cl^-^ [**(B)**, p = 0.0055], urinary Na^+^ [**(C)**, p < 0.0001], and urinary Cl^-^ [**(D)**, p = 0.0080], but not for urine osmolality [**(E)**, p = 0.3962]. No significant effect of genotype was detected [**(A)**, p = 0.4758; **(B)**, p = 0.7235; **(C)**, p = 0.5179; **(D)**, p = 0.6948; **(E)**, p = 0.3962], and no significant genotype x diet interaction was observed [**(A)**, p = 0.1409; **(B)**, p = 0.7235; **(C)**, p = 0.5801; **(D)**, p = 0.7063; **(E)**, p = 0.9184]. *Post-hoc* comparison p-values are indicated in the image. Data are presented as mean ± SD. Sample sizes ranged from n = 6 to n = 11 animals per group.

To assess renal function in claudin-3 knockout (KO) mice, we measured GFR by transcutaneous FITC-sinistrin clearance under normal sodium (NS) and low sodium (LS) diets. Representative curves of fluorescence decay for wild-type (WT) and KO mice under NS and LS diet are shown in **Figures 1A–D**. Following FITC-sinistrin administration, the fluorescence curve displayed three distinct phases: an initial peak corresponding to tracer distribution in the vascular compartment, a short plateau reflecting equilibration between plasma and tissues, and a monoexponential elimination phase driven by renal clearance. In accordance with the validated analytical procedure for this method, GFR calculation was based exclusively on the elimination phase through determination of the FITC-sinistrin elimination half-life (t_1/2_). Under NS conditions, claudin-3 KO mice exhibited a significantly reduced GFR compared with wild-type controls, both in absolute values (**Figure 1E**) and after normalization to body weight (**Figure 1F**), despite the absence of differences in plasma sodium and chloride concentration, urinary sodium and chloride excretion, or urine osmolality (**Figure 2**). In contrast, under LS conditions, GFR in claudin-3 KO mice paradoxically increased compared with wild-type animals, again both in absolute values (**Figure 1E**) and after normalization to body weight (**Figure 1F**), while electrolyte levels and urine osmolality remained similar between genotypes (**Figure 2**). Importantly, GFR remained unchanged between NS and LS conditions in wild-type mice, indicating that this bidirectional response was specific to claudin-3 deficiency. This unexpected bidirectional and sodium diet-dependent alteration in GFR strongly suggested that claudin-3 deficiency may contribute to TGF.

### Claudin-3 is expressed at the tight junctions of *macula densa* cells

Because GFR is tightly regulated by TGF and because claudin-3 is expressed in both the thick ascending limb (TAL) and the distal convoluted tubule (DCT), the two nephron segments at whose junction the *macula densa* is located (Sassi et al., 2026a), we hypothesized that claudin-3 is also expressed in *macula densa* cells.

To test this hypothesis, we performed immunofluorescence analysis on paraffin-embedded kidney sections from wild-type C57BL/6 mice.

**Figure 3:**
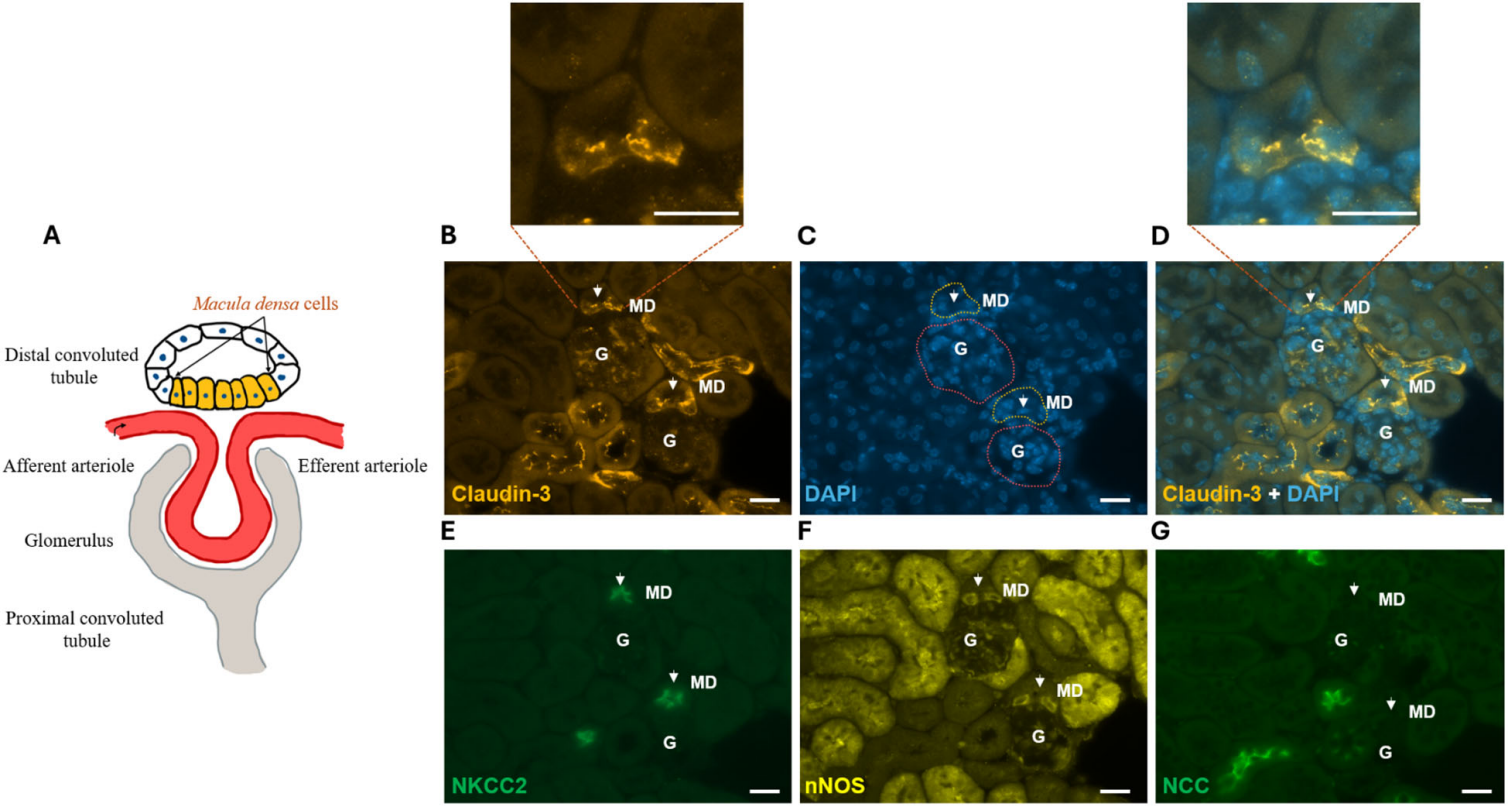
Claudin-3 is expressed at the tight junctions of *macula densa* cells. **(A)** Schematic illustration of the anatomical relationship between the glomerulus, the *macula densa* and the adjacent distal convoluted tubule at the TAL-DCT junction. **(B–G)** Representative immunofluorescence staining of serial kidney sections from wild-type C57BL/6 mice. **(B)** Claudin-3 staining (orange) in the renal cortex. **(C)** DAPI staining (blue) of the same section as in **(B)**. The upper parts of panels B and C are enlarged to better visualize Claudin-3 and DAPI labeling. **(D)** Merge of claudin-3 and DAPI staining of the same section. **(E)** NKCC2 staining (green). **(F)** nNOS staining (yellow). **(G)** NCC staining (green); the NCC-positive DCT is visible adjacent to the *macula densa*. G, glomerulus; MD, *macula densa*; arrow, *macula densa*. Scale bar: 20 µm.

The *macula densa* was identified based on its well-established anatomical, morphological, and molecular characteristics. As illustrated in the hand-drawn diagram (**Figure 3A**), it consists of a cluster of epithelial cells with densely packed nuclei located in direct contact with the glomerulus at the TAL-DCT junction and positioned immediately adjacent to the distal convoluted tubule. Molecularly, these cells are characterized by the expression of nNOS and NKCC2 and by the absence of NCC expression, which distinguishes them from the adjacent NCC-positive distal convoluted tubule segment (Bachmann et al., 1995; Obermüller et al., 1996; Bell et al., 2003).

Claudin-3 immunoreactivity was detected in a cluster of cells directly adjacent to the glomerulus and displaying the characteristic reticulated staining pattern of a tight junction protein (**Figure 3B**). Their anatomical position strongly suggested a *macula densa* localization. DAPI staining revealed the typical densely packed nuclei of these cells (**Figure 3C**), clearly visible in the merged claudin-3/DAPI image (**Figure 3D**), further supporting their identity as *macula densa* cells.

To confirm this localization, we performed additional immunostaining on serial sections using established *macula densa* markers. NKCC2 immunoreactivity was detected in the same cell cluster (**Figure 3E**). nNOS staining further confirmed *macula densa* identity, with signal strictly restricted to this cell cluster within the tubule, whereas the surrounding tubular epithelium of the same tubule remained nNOS−negative (**Figure 3F**).

Although nNOS expression has been predominantly described in *macula densa* cells, nNOS is also expressed at low levels in the proximal tubule, thick ascending limb, and collecting duct principal cells, with more limited expression in other segments (Han et al., 2006; Mount and Power, 2006). Within this context, its selective labeling of this cell cluster within the TAL–DCT transition remains a reliable histological criterion for *macula densa* identification. Notably, nNOS likewise labeled additional tubules that did not express Claudin-3 and therefore appeared to correspond to proximal tubules, consistent with the known absence of Claudin-3 in the proximal tubule and glomerulus (Sassi et al., 2026a). Importantly, NCC staining was absent from these cells, whereas an adjacent tubule was strongly NCC−positive (**Figure 3G**), consistent with the known absence of NCC in the *macula densa* and its expression in the neighboring DCT.

Taken together, the combination of claudin-3 immunoreactivity, densely packed nuclei, NKCC2 positivity, nNOS positivity restricted to the *macula densa* within the TAL-DCT transition, NCC negativity, and direct glomerular contact clearly identifies these cells as *macula densa* cells and demonstrates for the first time that claudin-3 is expressed at their tight junctions.

## Discussion

In this study, we report two novel findings. First, claudin-3 KO mice exhibit a diet-dependent, bidirectional alteration of GFR. Second, claudin-3 is expressed at the tight junctions of *macula densa* cells, as demonstrated by its colocalization with nNOS and NKCC2, together with densely packed nuclei, NCC negativity, and direct glomerular contact. Together, these observations identify claudin-3 as a previously unrecognized component of *macula densa* biology and provide a strong anatomical and physiological basis for further investigation of its role in glomerular hemodynamics.

The *macula densa*, a specialized epithelial structure located at the TAL-DCT junction, is not only a passive sensor of luminal NaCl concentration but an active signaling hub that integrates tubular information to regulate afferent arteriolar tone, renin secretion, and prostaglandin synthesis (Bell et al., 2003, 2009). Tight junction organization at the *macula densa* may critically influence the local ionic environment within the restricted interstitial space of the juxtaglomerular apparatus, thereby fine-tuning downstream TGF signaling. The presence of claudin-3 at *macula densa* tight junctions is anatomically consistent with its expression in both the TAL and DCT (Sassi et al., 2026a) and suggests that it may contribute to fine control of NaCl sensing, most likely by preventing luminal NaCl back-flux, at this strategic transition zone.

The GFR values observed in our study are comparable to those previously reported using the transdermal FITC-sinistrin technique (Arnoux et al., 2025; Correia de Sousa et al., 2025; Dalga et al., 2025). In wild-type mice, GFR remained stable under low-sodium diet, consistent with the preservation of normal TGF and in agreement with previous studies showing that physiological sodium restriction does not significantly alter GFR when TGF is intact (Udwan et al., 2017; Craigie et al., 2018). Under NS conditions, the absence of claudin-3 at the *macula densa* may alter local tight junction properties and modify NaCl handling at this site, resulting in an inappropriately reduced GFR. Conversely, under LS conditions, when luminal NaCl delivery is markedly reduced, claudin-3 deficiency may be associated with a paradoxical increase in GFR, which could reflect altered *macula densa*-dependent signaling during sodium deprivation. Interestingly, similar paradoxical increase in GFR under low-salt conditions has been reported in pathological conditions associated with impaired TGF, including diabetic nephropathy, where inappropriate hyperfiltration reflects defective *macula densa* signaling (Vallon et al., 2002, 2009).

This increased filtered sodium load could amplify the compensatory responses previously described in claudin-3 KO mice under LS, including the upregulation of ENaC subunits and claudins-4, -8, and -10 (Sassi et al., 2026a). However, while our findings are consistent with altered TGF, direct experimental evidence for a primary effect of claudin-3 on this pathway remains to be established. In particular, future studies will be required to determine whether claudin-3 deficiency affects key signaling events within the juxtaglomerular apparatus, including renin secretion, prostaglandin (particularly PGE2) production, afferent arteriolar responses to *macula densa* NaCl sensing, using approaches such as micropuncture or isolated perfused tubule preparations, as well as purinergic signaling and intracellular Ca^2+^ dynamics. Pharmacological blockade of TGF pathways may also help clarify the contribution of claudin-3 to this signaling cascade. Such studies will be essential to define the molecular mechanisms linking claudin-3 to TGF.

An additional aspect to consider is that the present study was performed exclusively in male mice. This choice was made to reduce biological variability and to maintain consistency with the original physiological characterization of the claudin-3 KO model. However, sex-dependent differences in renal hemodynamic regulation and TGF have been reported. In particular, testosterone has been shown to enhance TGF responsiveness through increased superoxide generation at the *macula densa*, whereas female mice exhibit enhanced *macula densa* NOS1β activity and nitric oxide-dependent attenuation of TGF, contributing to sex differences in GFR regulation and salt sensitivity (Fu et al., 2013; Zhang et al., 2020). Therefore, whether claudin-3 exerts similar effects on GFR regulation and *macula densa* physiology in female mice remains to be determined and should be addressed in future studies.

These findings open several important perspectives. Whether claudin−3 expression at the *macula densa* is regulated by dietary sodium intake or aldosterone and whether other claudins such as claudin−10, claudin−16, or claudin−19, which are expressed in the TAL and surrounding segments (Prot-Bertoye et al., 2021), are functionally relevant at the *macula densa* tight junctions remain important questions for future investigation.

From a translational perspective, dysregulation of claudin-3 at the *macula densa* may contribute to pathological conditions associated with altered TGF and sodium handling, including salt-sensitive hypertension, diabetic nephropathy, and acute kidney injury. More broadly, recent evidence highlighting the involvement of claudin-4 in renal sodium and chloride retention during nephrotic syndrome (Olivier et al., 2022) supports reconsidering tight junction proteins throughout the nephron not simply as structural barriers, but as dynamic regulators of tubular function and glomerular hemodynamics.

The identification of disease−causing mutations in claudin genes associated with inherited renal tubulopathies (Prot-Bertoye and Houillier, 2020; Alzahrani et al., 2021; Vall-Palomar et al., 2021; Sassi et al., 2026b) underscores the importance of claudins in the pathophysiology of electrolyte and water homeostasis, and highlights the potential for claudin−based mechanisms to contribute to glomerular hemodynamic and tubular sensing disorders. Together with evidence that claudin−3 and claudin−8 expression are hormonally regulated (Sassi et al., 2021, 2026a) and that claudin−8 functionally interacts with ENaC in the aldosterone−sensitive distal nephron (Sassi et al., 2020), these observations provide a strong conceptual framework to systematically investigating how claudin composition at the *macula densa* and along the TAL-DCT-collecting duct axis shapes tight junction organization, paracellular ion selectivity, tubuloglomerular signaling, and ultimately systemic blood pressure regulation.

## Conclusion

This study demonstrates for the first time that claudin-3 is expressed at the tight junctions of *macula densa* cells. Furthermore, claudin-3 deficiency is associated with a diet-dependent alteration in GFR, with reduced GFR under NS conditions and paradoxically increased GFR under LS conditions. These findings extend the role of claudin-3 beyond the collecting duct and identify the *macula densa* as a new site of claudin-3 expression. Although our data are consistent with a contribution of claudin-3 to *macula densa* function and possibly to TGF, direct functional evidence will be required to establish a direct role. Altogether, these results provide a new framework for understanding how tight junction organization may contribute to glomerular hemodynamics in both physiological and pathological states.

## Data Availability

The raw data supporting the conclusions of this article will be made available by the authors, without undue reservation.
